# Challenging human locomotion: stability and modular organisation in unsteady conditions

**DOI:** 10.1038/s41598-018-21018-4

**Published:** 2018-02-09

**Authors:** Alessandro Santuz, Antonis Ekizos, Nils Eckardt, Armin Kibele, Adamantios Arampatzis

**Affiliations:** 1 Department of Training and Movement Sciences, Humboldt-Universität zu Berlin, Berlin, 10115 Germany; 2 Institute for Sports and Sports Science, Universität Kassel, Kassel, 34121 Germany; 3 Berlin School of Movement Science, Humboldt-Universität zu Berlin, Berlin, Germany

## Abstract

The need to move over uneven terrain is a daily challenge. In order to face unexpected perturbations due to changes in the morphology of the terrain, the central nervous system must flexibly modify its control strategies. We analysed the local dynamic stability and the modular organisation of muscle activation (muscle synergies) during walking and running on an even- and an uneven-surface treadmill. We hypothesized a reduced stability during uneven-surface locomotion and a reorganisation of the modular control. We found a decreased stability when switching from even- to uneven-surface locomotion (p < 0.001 in walking, p = 0.001 in running). Moreover, we observed a substantial modification of the time-dependent muscle activation patterns (motor primitives) despite a general conservation of the time-independent coefficients (motor modules). The motor primitives were considerably wider in the uneven-surface condition. Specifically, the widening was significant in both the early (+40.5%, p < 0.001) and late swing (+7.7%, p = 0.040) phase in walking and in the weight acceptance (+13.6%, p = 0.006) and propulsion (+6.0%, p = 0.041) phase in running. This widening highlighted an increased motor output’s robustness (i.e. ability to cope with errors) when dealing with the unexpected perturbations. Our results confirmed the hypothesis that humans adjust their motor control strategies’ timing to deal with unsteady locomotion.

## Introduction

Although the mechanisms underlying human movement are not yet understood in their entirety, there is great amount of information on how we walk and run over even, solid surfaces^1–4^. Yet, daily-life locomotion is regularly taking place in more complex environments and conditions which imply facing an extraordinary amount of variables and interactions between them. Being able to effectively move over diverse terrain conditions has been integral part of the evolution of the genus *Homo*, not only to hunt and gather, but to escape predators and find mates as well^5,6^. It is well known that, for animals, one fundamental way to optimise locomotion is minimising the energy cost of motion^7–9^. However, optimisation of economy does not fully explain the movement strategies employed by humans and other animals^10^. Increased energy expenditure has been often found for walking and running on irregular natural or artificial terrain such as swamp, loam, stubble, grass, sand, snow, rubber, mountain trail and uneven-surface treadmill^11–20^. Studies showed that destabilising environments can decrease dynamic stability during walking^21–23^. Further, in presence of perturbations in the mediolateral direction, participants do not modify their velocity as a coping mechanism for stability, but rather alter spatiotemporal gait parameters^21,24^. It therefore seems that to overcome the non-predictability of uneven terrains, humans have to face constant changes in their locomotor patterns, which may affect the priorities of system.

The ability to move involves a flexible integration of peripheral sensory information into the central nervous system (CNS)^25^. Attempts to describe the biomechanics and energetics of balance control following single- and multiple-step perturbations have been made in both humans and animal models^9,18,19,26–35^. Though, very little is known about the neuromuscular strategies employed by the CNS to cope with external perturbations induced by continuous variations in terrain morphology. Lowering the energy cost of unsteady locomotor movements might not constitute the highest priority during locomotion and compromises to achieve, for instance, safety or perceived comfort could be of higher importance^10,33,36,37^. In the natural environment, animals must maintain dynamic stability when facing unexpected perturbations^33,38^. Locomotion over uneven terrain could challenge dynamic stability and, thus, the existing neuromuscular strategies. Alterations in the dynamic stability, continuously elaborated via sensory feedback information, provide a feed-forward drive to the CNS to implement the needed adjustments. To use a term derived from systems and software engineering, “robustness” defines “the degree to which a system or component can function correctly in the presence of invalid inputs or stressful environmental conditions”^39^. Likewise, a biological system is evolutionally robust when its characteristics can withstand perturbations or uncertainty^40^. In a similar fashion, we define “robustness” as the ability of the CNS to cope with unexpected perturbations or errors of execution. To better understand unsteady movement, the contribution of the neuromuscular circuit to the production and control of robust movement must be taken into account.

One of the most common hypothesis is that the CNS might simplify the production of movement by activating muscles in common patterns called synergies^41–43^. This strategy would avoid the separate activation of each muscle by linearly combining a small set of time-dependent commands^42,43^. Synergies can be seen as low dimensional units that, via descending or afferent pathways, produce a complex electromyographic (EMG) pattern in muscles^44,45^, creating a locomotor drive mediated by a certain amount of supraspinal control^46^. During walking, the same amount of basic activation patterns could be found in patients with spinal cord injury and in healthy participants at different speeds and gravitational loads^47^. Synergies similar to those found in humans at a spinal^47^ or muscular level can be observed also in the motor cortex of the primate and cat^48,49^. This suggests a high degree of cooperation within the central nervous system’s structure at all levels. We used an unsupervised learning method called non-negative matrix factorisation (NMF)^50^ for reducing the high dimensional EMG input into a small number of synergies.

A few studies already used the muscle synergies concept for investigating the modular control of balance following single-step^27^ and multiple-step^35^ perturbations during overground walking. Oliveira and colleagues^27^ showed that the modular organisation is preserved following a single-step perturbation during walking, while temporal activation patterns might differ. In another study^35^, Martino and colleagues found a preservation of the modular organisation after multiple-step walking on slippery ground and narrow beam. Contextually, they found an increase in the width (duration) of the muscle activity patterns during perturbed walking^35^, indicating a less accurate control. With “less accurate” we mean less precise in relation to the optimum or, in other words, including a higher degree of random errors that might move the system’s state more distant from the centre of the stability attractor^40^. The limitations of these studies, however, mainly lie in the small number of steps recorded, since the muscle synergies results might be influenced by the number of steps involved in the analysed data. Previous studies showed that a high number of steps (≥40) is important to increase the accuracy and precision of the muscle synergies analysis^51^. In order to increase the number of recorded steps and to accurately control the locomotion speed, the use of an uneven-surface treadmill is the most intuitive solution. Moreover, the continuously variable and unexpected perturbations would exclude any short-term predictive behaviour of the system and would help in the generalisation of the results. Exposure to continuously induced perturbations would challenge the system and might require alternative motor control strategies to be employed at the neuromuscular level. To support our analysis of the modular organisation of EMG activity, we investigated the spatiotemporal organisation of the alpha-motoneurons pools in the spinal cord^47^. While this method cannot help in characterising the central pattern generator circuitry, it is a useful tool to better understand the organisation of the motor output at a segmental level, rather than in terms of individual muscle activations.

We have limited knowledge on how humans choose from the available control strategies while moving through complex terrain. The purpose of the current study was to improve our understanding of the mechanisms underlying the neuromuscular control of human unsteady locomotion by investigating walking and running on an even- and an uneven-surface treadmill (Supplementary video 1). We hypothesised an increased instability in both walking and running on the uneven-surface treadmill compared to the even one. We expected that humans would respond to the increased instability by employing a different modular organisation of the neuromuscular output when walking and running on the uneven-surface treadmill. In particular, we expected a transfer from an accurate to a more robust neural control in unsteady locomotion.

## Methods

### Experimental protocol

Eighteen healthy and young adults were recruited (11 male, 7 female, height 176 ± 7 cm, body mass 71 ± 13 kg, age 24 ± 3 years, means ± s.d.). All the participants were regularly active and did not use orthotic insoles. None had any history of neuromuscular or musculoskeletal impairments, or any head or spine injury at the time of the measurements or in the previous six months. This study was reviewed and approved by the Ethics Committee of the University of Kassel (approval code E05201602). All the participants gave written informed consent for the experimental procedure, in accordance with the Declaration of Helsinki.

**Figure 1:**
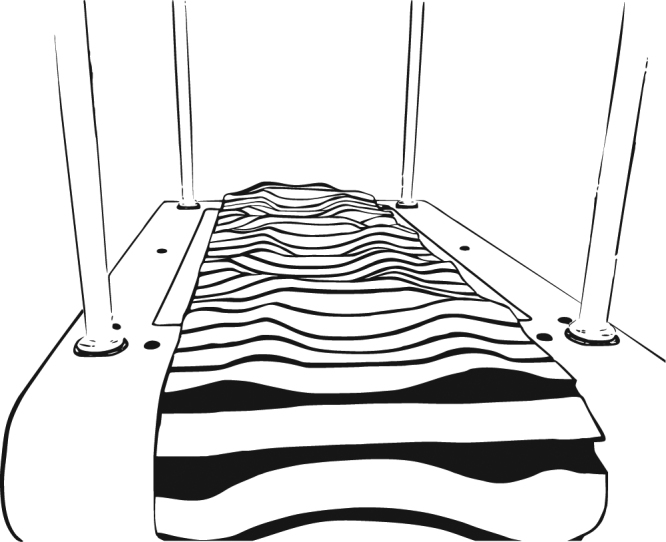
Sketch of the uneven-surface treadmill employed in this study.

The recordings were conducted on two treadmills: a standard one, equipped with an even-surface (ES) belt (Laufergotest, Erich Jäger, Würzburg, Germany) and one equipped with a custom-made uneven-surface (US) belt (Woodway®, Weil am Rhein, Germany, Fig. 1, Supplementary video 1). The US treadmill’s belt consisted of terrasensa® classic modules (Sensa® by Huebner, Kassel, Germany) aiming to simulate uneven ground conditions. The kinematics data were recorded through a six-camera motion capture system (Oqus 3+, Qualisys AB, Gothenburg, Sweden) operating at 300 Hz. The muscle activity of 13 ipsilateral muscles was recorded using a 16-channel wireless EMG system (myon m320, myon AG, Schwarzenberg, Switzerland), with a frequency of 1000 Hz.

The participants completed two different tasks on the ES and US treadmill, in random order: walking (shod, fixed velocity; 1.1 m/s female, 1.2 m/s male) and running (shod, fixed velocity; 2.0 m/s female, 2.2 m/s male). The difference in velocity between male and female have been chosen after a pilot study in which we estimated the average gender-specific comfortable running and walking velocity on the US treadmill. The participants were instructed to keep looking at a fixed spot in front of them and avoid looking at the treadmill’s belt.

### Gait cycle assessment

Ten reflective markers were placed bilaterally on the leg, foot and spine. Namely, the greater trochanter, the Achilles tendon insertion on the calcaneus, the dorsal margin of the fifth metatarsal head, the second, seventh and tenth thoracic and the second lumbar *vertebræ* were marked. The gait cycle breakdown was obtained from kinematic data. We used the information coming from the calcaneus and fifth metatarsal markers. These data were low-pass filtered using a 4^th^ order IIR Butterworth zero-phase filter with cut-off frequency of 50 Hz^52^. For estimating touchdown, we used the modified foot contact algorithm developed by Maiwald *et al*.^52^. For estimating lift-off, we developed the foot acceleration and jerk algorithm. The jerk algorithm searches for the global maximum of the fifth metatarsal vertical acceleration between two consecutive touchdown events to estimate the lift-off (LOe, where the “e” stays for “estimated”). This estimation, however, does not provide an accurate identification of the lift-off and needs some refinement. To get closer to the “real” lift-off timing, a characteristic minimum in the vertical acceleration (i.e. when the jerk equals zero) of the fifth metatarsal marker is identified in a reasonably small neighbourhood of the LOe. We found [LOe – 90 ms, LOe + 25 ms] for walking and [LOe – 50 ms, LOe + 100 ms] for running to be the sufficiently narrow intervals needed to make the initial lift-off estimation. Both approaches have been validated using force plate data (AMTI BP600, Advanced Mechanical Technology, Inc., Watertown, MA, USA) from 15 participants walking and running overground at six different velocities. True errors were of −8 ± 8 ms (−1.1% ± 1.0% of the stance phase) for touchdown and 12 ± 18 ms (1.6% ± 2.4%) for lift-off for walking and −1 ± 15 ms (−0.5% ± 5.4%) and −16 ± 23 ms (−5.7% ± 8.3%) respectively for running (means ± s.d.).

### Local dynamic stability assessment

Participants were allowed for an accommodation period of maximum 60 s^53^. During walking, two trials of 180 s were recorded for all participants, while two trials of 120 s were recorded during the running task. A 4^th^ order IIR Butterworth zero-phase filter with low-pass cut-off frequency of 20 Hz was applied to the computed coordinates of the four spine markers (i.e. the second, seventh and tenth thoracic and the second lumbar *vertebræ*). We adopted the maximum finite-time Lyapunov exponent (MLE) to assess the local dynamic stability of the human system during walking and running. The coordinates of the walking trials were downsampled to 100 Hz to improve computational performance. The vertical coordinates of these markers were then clustered to be used for further analysis and the calculation of the MLE. We calculated the MLE using the vertical coordinate data of the clustered spine markers, which we tested for stationarity^54,55^. Running on a treadmill restricts the movement at the anteroposterior direction due to the participants seeking to match the velocity of the treadmill and similarly, the treadmill width restricts the movement on the mediolateral direction. To avoid dependencies on step frequency, we identified the maximum number of shared steps (i.e. 0.5 of gait cycle) for all trials and participants^56^; 291 and 287 steps for walking and running, respectively. The coordinates of the data segments corresponding to the exact number of steps were then isolated for each trial. Following, the data segments were normalised to a uniform length based on the average number of data for each step, amounting ~16000 in walking and ~33000 data points in running. The high number of steps analysed ensured the reliability of our measurements^57^.

State space reconstruction was achieved through delay coordinate embedding^58,59^, for each point of the time series and its time-delayed copies as follows:

1 $$S(t)=[z(t),z(t+\tau ),\ldots ,z(t+(d-1)\tau )]$$

with *S*(*t*) being the *d*-dimensional reconstructed state vector, *z*(*t*) the input 1D coordinate series, *τ* the time delay and *d* the embedding dimension. Time delays were calculated for each time series from the first minimum of the mutual-information curve, based on the Average Mutual Information function^60^. The number of embedding dimensions was extracted through a Global False Nearest Neighbours analysis for each time series, with a threshold of one per thousand data points^61^.

Different values of *τ* and *d* can yield very different state-space reconstructions^54,62,63^. It is therefore suggested that optimised values of *τ* and *d* are necessary to best represent a dynamical system^64^. In the current study time delays were individually chosen for each participant^64^, by first calculating the optimal delay of the four time-series (two trials on the even and two on the uneven surface) and using the averaged value. Time delays were in the range of 17–20 data points (~0.34 of the average step) in the walking task and 36–40 data points (~0.33 of the average step) in the running task.

Following the reconstruction of the times series, the Rosenstein algorithm was used to compute the average exponential rate of divergence by calculating the linear distance of each point’s trajectory divergence to its closest trajectory^65,66^. The MLE were then calculated from the slope of the linear fit in the resulting divergence curves from 0 to 1 step. Analysis of the data was performed on MATLAB 2014b (Mathworks Inc., Natick, United States).

### Spinal motor output assessment

For each condition, the muscle activity of the following 13 ipsilateral (right side) muscles was recorded: gluteus medius (ME), gluteus maximus (MA), tensor fasciæ latæ (FL), rectus femoris (RF), vastus medialis (VM), vastus lateralis (VL), semitendinosus (ST), biceps femoris (long head, BF), tibialis anterior (TA), peroneus longus (PL), gastrocnemius medialis (GM), gastrocnemius lateralis (GL) and soleus (SO). We recorded two trials of 60 s for each condition and analysed the first 50 gait cycles of each acquisition. The EMG signals were high-pass filtered and then full-wave rectified and low-pass filtered using a 4^th^ order IIR Butterworth zero-phase filter with cut-off frequencies 50 Hz (high-pass) and 20 Hz (low-pass for the linear envelope) using R v3.4.1 (R Found. for Stat. Comp.). The amplitude was normalised to the maximum activation recorded for each participant across all conditions^43,67,68^. Each gait cycle was then time-normalised to 200 points^69^, assigning 100 points to the stance and 100 points to the swing phase.

For the spinal motor output characterisation, we mapped the EMG activity onto the estimated rostrocaudal location of alpha-motoneurons (MNs) pools in the segments from the second lumbar vertebra (L2) to the second sacral vertebra (S2) of the spinal cord^47,70,71^. The contribution of each muscle to the total estimated activity of the spinal segments was implemented using the myotomal charts developed by Kendall *et al*.^72^. This method shows the organisation of the efferent MNs network directed to the muscles, assuming a common spinal topography among the investigated participants. The motor output of each spinal segment *S*_*j*_ was estimated using the Equation (2) introduced by La Scaleia *et al*.^70^:

2 $${S}_{j}=\frac{{\sum }_{i=1}^{{m}_{j}}(\frac{{k}_{ji}}{{n}_{i}}\times EM{G}_{i})}{{\sum }_{i=1}^{{m}_{j}}(\frac{{k}_{ji}}{{n}_{i}})}\times M{N}_{j}$$

where *m*_*j*_ are the muscles innervated by each segment, *n*_*i*_ is the number of spinal levels that innervate the *i*^th^ muscle, *k*_*ij*_ is a weighting coefficient specific to each muscle and spinal segment (e.g. *k*_*ij*_ = 1 or *k*_*ij*_ = 0.5 if *S*_*j*_ is a major or minor MN source, respectively) and *EMG*_*i*_ is the normalised recorded EMG, specific for each participant and trial^70,72^. This approach accounts for size differences at each spinal level in every *MN*_*j*_ pool.

### Modular organisation assessment

Muscle synergies data were extracted through a custom script^73^ (R v3.4.1, R Found. for Stat. Comp.) using the classical Gaussian NMF algorithm^50,73,74^ from the first 50 gait cycles of each acquisition^51^. EMG data were pre-processed using the same filtering conditions reported in the previous paragraph and each gait cycle was time-normalised to 200 points^69^, assigning 100 points to the stance and 100 points to the swing phase^74^. The *m* = 13 time-dependent muscle activity vectors were grouped in an *m* *×* *n* matrix *V* (*n* equal to 10000), factorised such that *V≈V*_*R*_ *=* *WH*. *V*_*R*_ represents the new reconstructed matrix, which approximates the original matrix *V*. The motor primitives^73,75^ matrix *H* contained the time-dependent coefficients of the factorisation with dimensions *r* *×* *n*, where *r* represents the minimum number of synergies necessary to reconstruct the original signals (*V*). The motor modules^73,76^ matrix *W* with dimensions *m* *×* *r*, contained the time-invariant muscle weightings. *H* and *W* described the synergies necessary to accomplish a movement. The update rules for *H* and *W* are presented in Equations (3) and (4).

3 $${H}_{i+1}={H}_{i}\frac{{{W}_{i}}^{T}V}{{{W}_{i}}^{T}{W}_{i}{H}_{i}}$$

4 $${W}_{i+1}={W}_{i}\frac{V{({H}_{i+1})}^{T}}{{W}_{i}{H}_{i+1}{({H}_{i+1})}^{T}}$$

The limit of convergence was reached when a change in the calculated *R*^2^ between *V* and *V*_*R*_ was smaller than the 0.01% in the last 20 iterations^73,77^. This was done for a number of synergies successively increased from 1 to 10. The computation was repeated 10 times for each synergy, each time creating new randomised initial matrices *H* and *W*, in order to avoid local minima^73,78^. The solution with the highest R^2^ was then selected for each of the 10 synergies.

**Figure 2:**
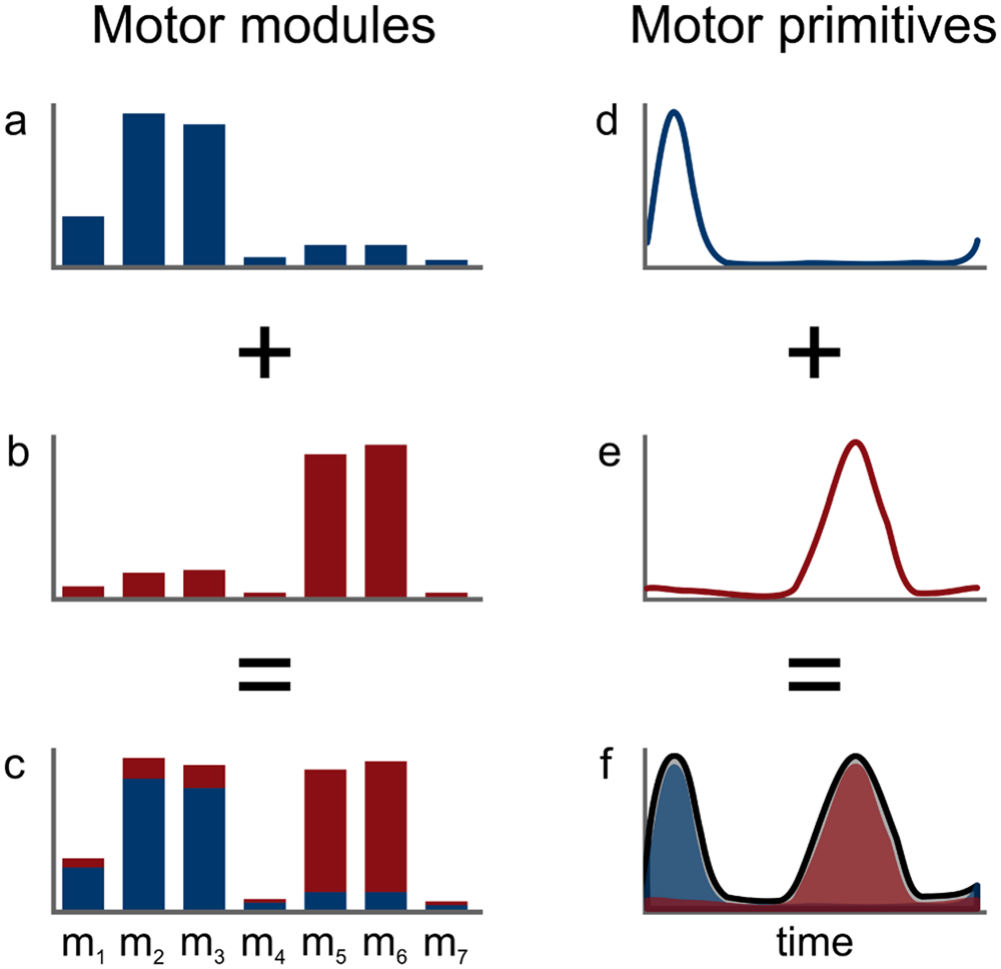
Example of two fundamental synergies combined into one. The histograms (**a** and **b**) represent the two fundamental sets of motor modules for seven muscles. The curves (**d** and **e**) show the two respective primitives, with arbitrary x- and y-axis units. The combined motor modules and primitives are presented in panels (c and f), respectively.

To choose the minimum number of synergies required to represent the original signals, the curve of *R*^2^ values versus synergies was fitted using a simple linear regression model, using all ten synergies. The mean squared error^73^ was then repeatedly calculated, each time removing the lower synergy point, until only two points were left or until the mean squared error fell below 10^−5^. The aforementioned procedure allowed us to extract fundamental and combined synergies from the raw EMG data. A fundamental synergy can be defined as an activation pattern whose motor primitive shows a single peak of activation^73,74^. When two or more fundamental synergies are blended into one, a combined synergy appears. Combined synergies were excluded from the analysis. An example of combined synergies is reported in Fig. 2.

### Metrics for comparison of curves

We evaluated the centre of activity (*CoA*) and full width at half maximum (FWHM) for the resulting curves of the extracted spinal maps and motor primitives (matrix H) in both conditions and types of locomotion. The *CoA* was defined as the angle of the vector (in polar coordinates) that points to the centre of mass of that circular distribution^69,74^. The polar direction represented the gait cycle’s phase, with angle 0 ≤ *θ*_*t*_ ≤ 2π. The following equations define the *CoA*:

5 $$A=\sum _{t=1}^{p}(\cos \,{\theta }_{t}\times {P}_{t})$$

6 $$B=\sum _{t=1}^{p}(\sin \,{\theta }_{t}\times {P}_{t})\,$$

7 $$CoA=\arctan (B/A)$$

where *p* is the number of points of each gait cycle (*p* = 200) and *P* is the activation vector. The FWHM was calculated as the number of points exceeding each gait cycle’s half maximum, after subtracting the gait cycle’s minimum^69,74^. The first 50 gait cycles of each acquisition were selected for analysis. The *CoA* and FWHM were analysed step-by-step and then averaged for stance and swing distinctively for spinal maps and over the whole gait cycle for the motor primitives.

### Statistics

To evaluate the differences in MLE and gait parameters between ES and US locomotion, we used a one-way ANOVA with repeated measures or non-parametric Friedman test in case the normality assumptions on the residuals were not satisfied. To investigate *CoA* and FWHM of the spinal motor output, we employed a two-way ANOVA with repeated measures, followed by a Tukey *post-hoc* analysis with false discovery rate *p-value* adjustment (the independent variables being the condition, i.e. ES or US, and the spinal segment). The same statistics was used to assess differences between the motor modules, using the muscles and the conditions (ES or US) as independent variables. To evaluate the similarities between the fundamental motor primitives of the ES and US conditions, we used the coefficient of determination *R*^2^. The analysis was conducted as follows: first, we calculated the similarity between the pairs of trials recorded during ES and US locomotion (i.e. the repeatability level when comparing two trials of the same condition)^73,74^. Then, we statistically compared the similarity between trials of different conditions (ES and US locomotion) with the repeatability values. To do so, we used a one-way ANOVA with repeated measures or non-parametric Friedman test in case the normality assumptions on the residuals were not satisfied. Type A uncertainty was expressed as ${u}_{A}=s/\surd n$. All the significance levels were set to α = 0.05 and the statistical analyses were conducted using R v3.4.1 (R Found. for Stat. Comp.).

### Data availability

The datasets generated and analysed during the current study are available from the corresponding author on reasonable request.

## Results

### Gait parameters

The contact times in walking did not differ significantly (p = 0.539) between ES (674 ± 37 ms) and US (668 ± 49 ms). In running, there was a significant decrease (p < 0.001) in contact time when switching from ES (353 ± 50 ms) to US (324 ± 41 ms). Similarly, cadence did not change in walking (p = 0.589, 109 ± 5 and 110 ± 7 steps/min for ES and US, respectively), while in running it increased significantly (p < 0.001) when transitioning from ES (155 ± 7 steps/min) to US (160 ± 10 steps/min, means ± s.d.).

### Local dynamic stability

**Figure 3:**
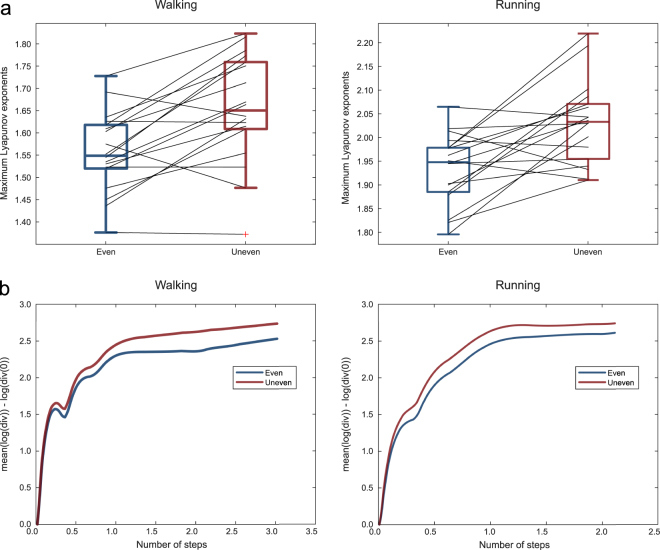
Boxplots depicting the maximum Lyapunov exponent values for even and uneven surface in both walking and running with the black lines indicating individual changes between conditions (**a**). Averaged over all participants mean logarithmic divergences of the trajectories between even and uneven surface in walking and running. A faster divergence indicates worse dynamic stability (**b**).

The MLE were significantly higher in the US condition in both walking (ES: 1.557 ± 0.087, US: 1.655 ± 0.119, p < 0.001) and running (ES: 1.936 ± 0.072, US: 2.031 ± 0.085, p = 0.001, means ± s.d.), evidencing an increased instability in the US (Fig. 3).

### Spinal motor output

**Figure 4:**
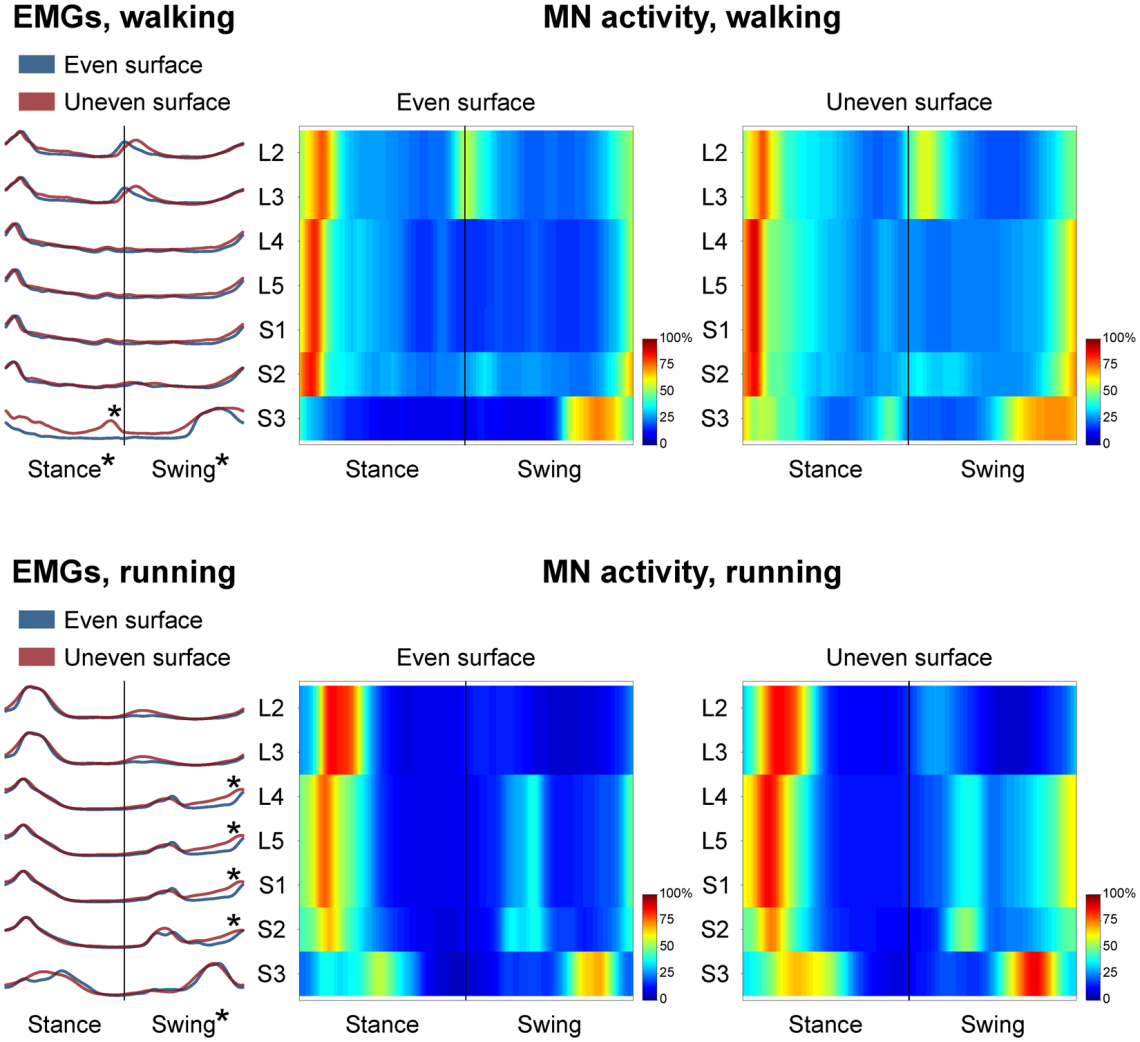
The average spatiotemporal spinal motor outputs are presented for even and uneven surface walking and running, normalised in amplitude to the maximum of each segment. These curves have been obtained by mapping each of the 13 muscle activations onto the relevant spinal segment (lumbar from L2 to L5 and sacral from S1 and S3). Asterisks denote significant differences in the full width at half maximum of the mapped EMGs between even and uneven surface locomotion. The two level plots show the average alpha-motoneurons activity for each condition, giving additional information about the absolute activation level (normalisation to the maximum of each condition). The stance and swing phases have been temporally normalised to the same amount of data points (100 each). Values are the means across all subjects and all trials.

Figure 4 depicts the average spinal motor output for ES and US walking and running. The two-way ANOVA identified statistically significant differences (p < 0.001, Table 1) in the *CoA* of the mapped EMG activities when comparing ES and US locomotion only for the stance phase of the walking cycle. The *post-hoc* analysis showed a significant contribution (p < 0.001) of the S3 spinal segment. The FWHM of the mapped EMG activities was significantly different between ES and US conditions for the stance as well as the swing phase of walking (p = 0.002 and p = 0.001 respectively, Table 2). For running, the FWHM was significantly different (p < 0.001) only in the swing phase (Table 2). The *post-hoc* analysis evidenced significantly greater FWHM values (p < 0.001) in the US condition in the S3 spinal segment for walking (stance phase). For running, the segments L4, L5, S1, S2 in the swing phase were involved instead (Table 2).

**Table 1:** Differences between even and uneven surface in the centre of activity (*CoA*) of the EMG activities mapped onto the estimated rostrocaudal location of the spinal cord (segments L2 to S3).

Segment	Walking			Running	
	Stance *p* < 0.001*		Swing *p* = 0.110	Stance *p* = 0.526	Swing *p* = 0.648
	Δ_E,U_	*p*-*value*	Δ_E,U_	Δ_E,U_	Δ_E,U_
L2	+1.4%	0.783	−6.8%	+0.7%	−7.1%
L3	+1.4%	0.783	−6.8%	+0.7%	−7.1%
L4	+2.9%	0.416	−0.8%	+0.0%	+4.8%
L5	+2.9%	0.416	−0.8%	+0.0%	+4.8%
S1	+2.9%	0.416	−0.8%	+0.0%	+4.8%
S2	+4.0%	0.285	−0.5%	−0.7%	+3.9%
S3	+*13.7*%	<0.001*	−3.6%	−3.6%	+0.8%

Positive differences (Δ_E,U_ > 0) denote bigger values in the uneven surface condition, whereas negative differences imply lower values.

**Table 2:** Differences between even and uneven surface in the relative full width at half maximum (FWHM) of the EMG activities mapped onto the estimated rostrocaudal location of the spinal cord (segments L2 to S3).

Segment	Walking				Running		
	Stance *p* = 0.002*		Swing *p* = 0.011*		Stance *p* = 0.119	Swing *p* < 0.001*	
	Δ_E,U_	*p*-*value*	Δ_E,U_	*p*-*value*	Δ_E,U_	Δ_E,U_	*p*-*value*
L2	−0.6%	0.610	+2.0%	0.279	+1.2%	+0.1%	0.070
L3	−0.6%	0.610	+2.0%	0.279	+1.2%	+2.7%	0.070
L4	+1.2%	0.252	+1.8%	0.315	+0.8%	+*2.7*%	<0.001*
L5	+1.2%	0.252	+1.8%	0.315	+0.8%	+*7.3*%	<0.001*
S1	+1.2%	0.252	+1.8%	0.315	+0.8%	+*7.3*%	<0.001*
S2	+0.1%	0.942	−1.0%	0.552	−1.0%	+*4.4*%	0.003*
S3	+*5.9*%	<0.001*	+2.5%	0.133	+3.4%	+*2.0*%	0.172

Positive differences (Δ_E,U_ > 0) denote bigger values in the uneven surface condition, whereas negative differences imply lower values.

### Modular organisation

**Figure 5:**
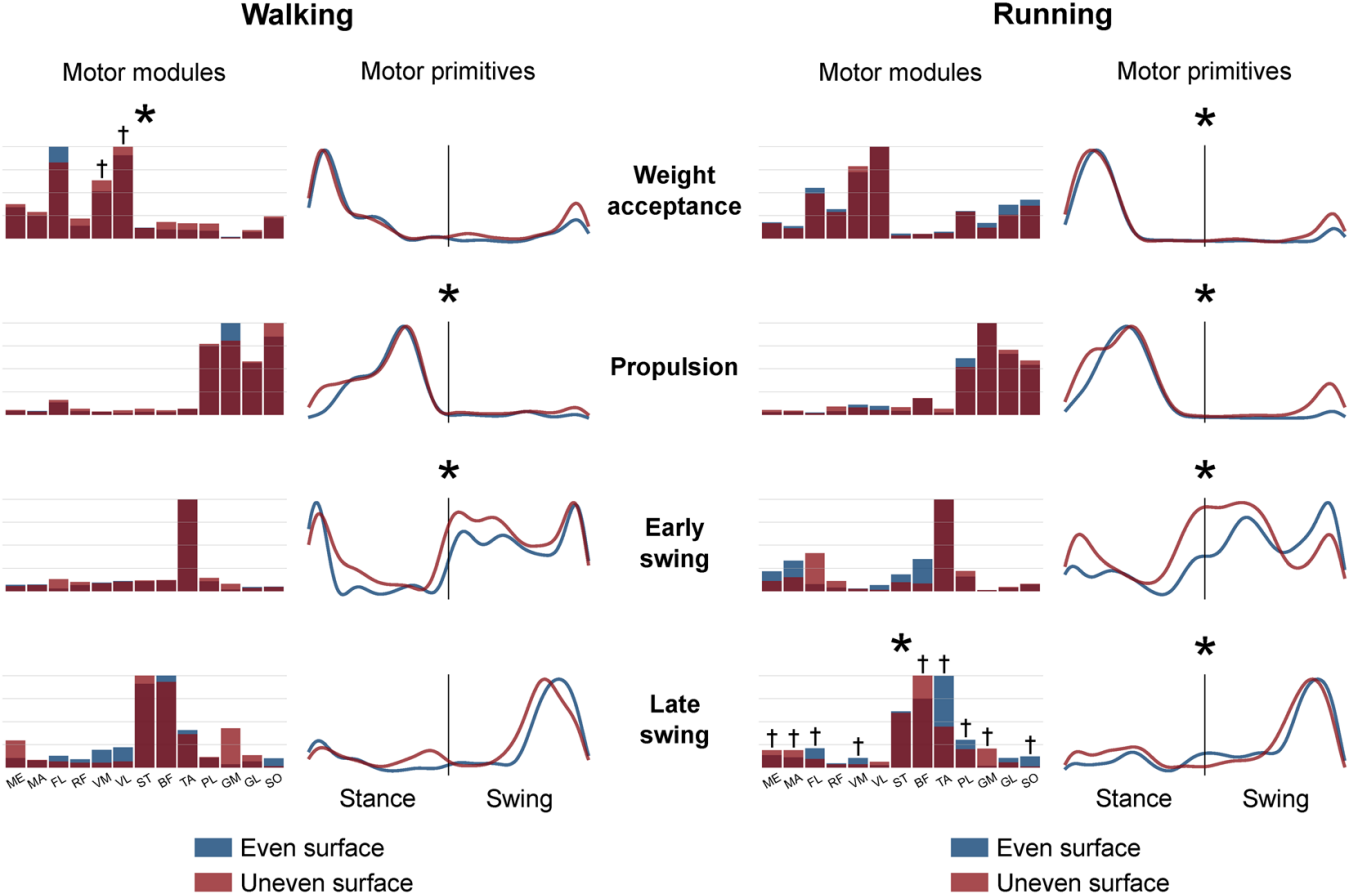
Average motor modules and motor primitives of the four fundamental synergies for walking and running on even and uneven surface. The motor modules are presented on a normalised y-axis base. For the motor primitives, the x-axis full scale represents one gait cycle (stance and swing normalised to the same amount of points and divided by a vertical line) and the y-axis the normalised amplitude. Asterisks denote significant differences between even and uneven surface locomotion. Daggers denote results of the *post-hoc* analysis.

The minimum number of synergies necessary to sufficiently describe the measured EMG-activity during walking and running was not significantly different between ES and US in either walking (3.8 ± 0.6 for ES and 3.9 ± 0.5 for US, p = 0.665) or running (3.5 ± 0.5 for ES and 3.5 ± 0.7 for US, p = 0.743, means ± s.d.). In both locomotor activities, four fundamental activation patterns could be identified (Fig. 5). The four fundamental synergies extracted during ES and US walking and running were associated with temporally different phases of the gait cycle. The first synergy (peak at ~12% and ~21% of the stance phase for walking and running, respectively) functionally referred to the body weight acceptance, with a major involvement of knee extensors and glutei. The second synergy (peak at ~70% and ~46% of the stance phase for walking and running, respectively) described the propulsion phase, to which the plantarflexors mainly contributed. The third synergy (peak at ~8% and ~30% of the swing phase for walking and running, respectively) identified the early swing, showing the involvement of foot dorsiflexors. The fourth and last synergy (peak at ~75% and ~80% of the swing phase for walking and running, respectively) reflected the late swing and the landing preparation, highlighting the relevant influence of knee flexors and foot dorsiflexors.

In walking, the similarities between the ES and US motor primitives of the propulsion and early swing synergies were significantly lower than the intraday repeatability threshold (Fig. 5, Table 3). In running, the motor primitives of all synergies were found to be significantly different between ES and US locomotion (Fig. 5, Table 3). The motor modules of ES and US locomotion exhibited significant differences in the weight acceptance synergy for walking (p = 0.001) and in the late swing synergy for running (p = 0.046, Fig. 5).

**Table 3:** Motor primitives’ similarities, indicated as R^2^_E,U_, between even and uneven surface walking and running as mean of intraday repetitions.

		Motor primitives		
		R^2^_E,U_	R^2^_E,U_ intraday	p-value
Weight acceptance	Walking	0.78 ± 0.20	0.89 ± 0.09	0.055
	Running	*0.73* ± *0.27*	*0.94* ± *0.09*	0.001*
Propulsion	Walking	*0.73* ± *0.20*	*0.89* ± *0.10*	0.004*
	Running	*0.70* ± *0.21*	*0.95* ± *0.03*	<0.001*
Early swing	Walking	*0.05* ± *0.83*	*0.79* ± *0.13*	<0.001*
	Running	*−0.34* ± *0.76*	*0.74* ± *0.65*	<0.001*
Late swing	Walking	*0.72* ± *0.18*	*0.85* ± *0.17*	0.059
	Running	*0.73* ± *0.16*	*0.95* ± *0.08*	<0.001*

The intraday repeatability values are reported as mean of four trials (two on the even and two on the uneven surface). Means ± Type A uncertainty. The *p-values* were calculated by comparing the R^2^ between even and uneven and the R^2^ for intraday trials.

The *CoA* of the motor primitives for the propulsion, early swing and late swing synergies moved significantly earlier in time (p < 0.05) in US compared to ES locomotion (Table 4, the only exception being the late swing phase in running). Further, we found an increase of the FWHM in the US compared to ES condition of the primitives related to early and late swing in walking and to the weight acceptance and propulsion in running (Table 4).

**Table 4:** Differences between even and uneven surface walking and running in the centre of activity (*CoA*) as well as in the relative full width at half maximum (FWHM) of motor primitives.

		Motor primitives			
		CoA		FWHM	
		Δ_E,U_	*p*-*value*	Δ_E,U_	*p*-*value*
Weight acceptance	Walking	−0.6%	0.478	+4.0%	0.398
	Running	−1.1%	0.112	+13.6%	0.006*
Propulsion	Walking	*−2.5%*	0.002*	+8.2%	0.180
	Running	*−3.8%*	<0.001*	+*6.0%*	0.041*
Early swing	Walking	*−6.7%*	0.002*	+*40.5%*	<0.001*
	Running	*−14.1%*	<0.001*	+10.0%	0.202
Late swing	Walking	*−2.7%*	0.001*	*+7.7%*	0.040*
	Running	+*1.6%*	0.008*	*+3.0%*	*0.212*

Positive differences (Δ_E,U_ > 0) denote bigger values in the uneven surface condition, whereas negative differences imply lower values.

## Discussion

The current study examined the neuromuscular control of normal and perturbed movement during walking and running. We hypothesized a decrease in the dynamic stability and a transfer from an accurate (i.e. mature, functionally fine-tuned) to a more robust (i.e. able to cope with unexpected errors) motor control during US locomotion. We found higher values in the MLE (i.e. higher instability) and a widening in the motor primitives (i.e. alterations in the temporal structure of motor control) and spinal motor output, which evidenced an increased robustness^35,69^ of the system during US locomotion. The findings confirmed our hypotheses and demonstrated the use of a consistent set of neural control elements during perturbed steady state locomotion, but with modifications of the basic activation patterns. This indicated a transition from an accurate to a more robust movement control in the presence of continuously variable perturbations.

The MLE quantifies how the system responds to small internal perturbations^56^, revealing the ability of the system to maintain stability and detects neuromuscular control errors in achieving it^66,79^. Increased MLE correspond to a more chaotic and unstable dynamical system^54,62^. In our study the MLE increased ~6% in walking and ~5% in running on the US providing evidence for a clear reduction of stability during perturbed locomotion. Previous studies in walking found an increase of 9% in the MLE in patients with focal cerebellar lesion^80^ and 21% increase in patients with moderate neurological gait disorders^81^, while in running an increase of 2% in the MLE was found during the transition from shod to barefoot^64^. The introduced perturbations, unpredictable and continuously variable in amplitude, interfered with the normal locomotor patterns affecting the neuromuscular control. Based on our findings, the US locomotion resulted in a decreased dynamic stability and likely drove the system to adjust the motor output for robustness. The wider shape of the motor primitives during US locomotion was coupled with a temporal shift in the *CoA*. A similar tendency could be observed as well in the estimated spinal motor output, supporting the idea of a temporal widening also in terms of segmental organisation. Specifically, the spinal motor output in walking was significantly wider especially in the stance phase (but in the swing phase as well), mainly due to the innervations of the muscle *biceps femoris* (spinal segment S3). During running, the width of the spinal motor output was significantly different only in the swing phase, but for almost all segments and, most importantly, due to the contributions of all muscles’ innervations. This result fits with the *post-hoc* analysis conducted on the motor modules of this locomotion type. However, both locomotion conditions showed a similar modular organisation, since four synergies were sufficient to describe the motor task in both ES and US locomotion. These findings provide evidence that the central nervous system uses a consistent set of neural control elements with a flexible temporal recruitment to create safe locomotion in the presence of continuous perturbations during walking and running. The kind of perturbation induced by the US treadmill used in this study was uninterrupted over the acquisition time, thus creating a new perturbation at each step. Since the participants were asked not to look at their feet, we can exclude any proper predictive behaviour (i.e. experience-based prediction of expected perturbations^34^) in all conditions. Nevertheless, we can expect a certain level of anticipation utilised to cope with the potential perturbations. However, the participants expected continuously variable perturbations and therefore might have been able to create anticipatory muscle activation patterns driven by knowledge and prior experience with the potential perturbation. The main alterations in the modular organisation of the less stable (US) locomotion occurred in the basic activation patterns (motor primitives) rather than in the number of muscle synergies or the structure of the motor modules. Muscle synergies are coordinated patterns of muscle activity that aim to create functional motor output from the interplay of spinal and supraspinal interactions with the environment^43,45^. Synergies might be expressed via motor circuits in the cortex, brainstem and spinal cord^46,49,82^. There is indirect evidence that intrinsic networks of spinal interneurons might be involved in rhythm generation, left-right alternation and flexor-extensor interaction^28,83–89^. Proofs that a finer, time-dependent tuning of novel or learned elementary spinal commands might be of a supraspinal nature, have been found in the cat^48,90^ and monkey^49,91,92^. This suggests that the descending commands (i.e. motor primitives) from the brainstem and motor cortex modulate spinal motor modules^28,45^. Therefore, the widening of the motor primitives indicates a relevant contribution of supraspinal structures in the control of perturbed locomotion.

Previous studies found shorter, faster and wider steps in response to destabilising environments during walking^21,93^. Shorter times to plan and execute movements decrease their accuracy^24,94^. In our study, similar findings in contact times and cadence (i.e. shorter contact times and higher cadence) were present only during running, possibly due to the fact that the perturbations were continuously induced and affected all planes of motion. This partial inconsistency could be further explained by the magnitude of the induced perturbations, which in our study was not quantifiable and possibly too low to cause modifications in the spatiotemporal parameters of walking. However, when analysing the step-to-step variability, we could find a tenfold increase in the contact times variance in walking (p < 0.001). To a minor extent, but still significantly (p = 0.001), this variability was also present in running, where variances increased 2.8 times. However, only in running we could demonstrate a decrease in the average contact times. This displays that the choice of a proper timing of execution is of crucial importance for managing external perturbations. When the phasing of events is less predictable (i.e. stereotyped to an extent that can be managed by a standard set of anticipatory spatiotemporal commands), a loss in accuracy can be expected. Broader basic activation patterns and EMG profiles have already been associated with inaccuracy and variability in motor control as well as with higher metabolic cost in different gait conditions^18,19,35,69^. Therefore, the system maintains successful locomotion by making up for a decrease in accuracy with an increase in robustness, which is reflected in the widening of the motor output.

During development, the locomotor activity undergoes adaptations which are linked to a functional reorganisation of the motor output^69^. As recently reported from Cappellini *et al*., typically developing children show, during walking, a gradual reduction in the FWHM of motor primitives associated with maturation (i.e. an improvement in accuracy)^69^. Conversely, cerebral palsy children show broader motor primitives compared to typically developing children at the same age^69^. Moreover, in children affected by cerebral palsy, the narrowing of the motor primitives with age is lacking, despite a comparable structure of motor modules^69^. Analogously, a widening of the motor primitives can be found in adult patients with cerebellar ataxia and in healthy adults walking on a narrow beam and on slippery ground^35^. The prolongation of the basic activation patterns might reflect the system’s need of adding robustness to maintain functionality and overcome continuous perturbations. If we think of motor primitives’ widening as a modification of the states of the system, we can assume that functionality is maintained, but the strategy to achieve it has slightly changed. In fact, it is commonly accepted that a system can achieve robustness by either returning to its current attractor or by moving to a new attractor which is good enough to maintain the system’s functions^40^. When a modification in the system’s state happens and the system is able to return to its original attractor, there is a so-called “robust adaptation”^40^. Furthermore, it is also true that, in case of a transition to a new attractor, the switch must preserve enough robustness in order to allow the system for consistent and adequate responses to perturbations^40^. This is a crucial feature of robust adaptation, since it allows the system to maintain specific functionalities with different, flexibly selected modes of operations^40^. Under a control systems’ perspective, it is known that fuzzy control systems decrease robustness in order to increase optimality and performance^95^. Looking at the widening of the motor primitives in the US condition, it is in fact possible to note that two chronologically adjacent primitives (e.g. weight acceptance and propulsion synergies during running or early swing and late swing during walking) are overlapping more than in ES locomotion. This increases the fuzziness of the temporal boundaries, thus creating a “buffer” of motor control that allows for shifting more easily from one synergy (or gait phase) to the other, increasing the robustness and contextually decreasing optimality and performance^95^.

In our study, the widening found in the spinal maps and motor primitives was associated to the less practiced and more unstable of the two tasks (i.e. US walking and running), indicating a less refined timing and duration of the motor output. The requirement to cope with continuously variable perturbations in order to maintain dynamic stability challenges the neural system’s control of locomotion. As such, it may reduce the need for accurate neural control, prioritising the search for robustness. The widening of the motor primitives in US walking and running was common among all participants. Concerning motor modules, we could confirm a significant alteration only for those related to the weight acceptance synergy in walking and to late swing in running. This provides an indication of a certain degree of conservation in the basic modular structure. Furthermore, our data confirmed that it is possible to describe with the same number of synergies not only both walking and running^1^, but ES and US locomotion as well. This observation provides additional support to the idea that the CNS may be able to modulate existing synergies in order to face different locomotion conditions.

During locomotion, the muscle activation patterns are characterised by a flexible modulation dependent on several external and internal factors. It has been shown by Akay *et al*. that the absence of proprioceptive sensory feedback from muscle spindles and Golgi tendon organs deteriorates the coordination of walking and swimming in mice^89^. A flexible modulation of the motor output has been found in humans undergoing changes in the mechanical demands of walking in both *in vivo*^47^ and *in silico* models^96^. Biewener and Daley proposed that mechanical effects are likely to be the predominant motor controllers at high locomotion velocities (e.g. running), since feedback delays might be destabilising^9^. Yet, at lower gait velocities like in walking, proprioceptive sensory feedback might provide superior contribution to stability control^9^, a concept that has been confirmed by scale studies on the sensorimotor responsiveness in the giraffe^97^. As mentioned above, we could not exclude some anticipatory behaviour in our participants to cope with the ground changes when walking or running on the US treadmill. The widening of the weight acceptance and propulsion primitives (i.e. alterations directly after touch down) in US running indicates an amplified anticipatory adjustment compared to walking (widening in the early and late swing primitives). On the one hand, appropriate anticipatory adjustments during perturbed locomotion at high velocity could have forced the system to rely more on the intrinsic mechanical response than on the sensory feedback during the stance phase. On the other hand, the widening in walking was present only in the two swing phases (i.e. when the system stands on one leg). This suggests an increase in the robustness of the swinging leg’s neuromuscular control when the contralateral limb is reinforcing the proprioceptive sensory feedback by exchanging forces with the ground. Thus, our findings would support the speed-dependent strategies for controlling locomotion hypothesized by Biewener and Daley.

In conclusion, the findings of the current study provide evidence that humans adjust their motor control strategies when walking or running over uneven terrain. We found that the changes in terrain morphology decreased the dynamic stability of the system, resulting in a temporal rearrangement (widening) of the motor primitives’ shape, despite a general preservation of the motor modules’ structure. The widening indicates an increase in the system’s robustness to deal with the induced perturbations. These observations suggest that supraspinal processes might be largely involved in the control of unsteady locomotion, with possible differences in the utilisation of proprioceptive sensory feedback between walking and running.

## Electronic supplementary material

Video 1
